# Accuracy of narrow band imaging for detecting the malignant transformation of oral potentially malignant disorders: A systematic review and meta-analysis

**DOI:** 10.3389/fsurg.2022.1068256

**Published:** 2023-01-06

**Authors:** You Zhang, Yuqi Wu, Dan Pan, Zhenyu Zhang, Lu Jiang, Xiaodong Feng, Yuchen Jiang, Xiaobo Luo, Qianming Chen

**Affiliations:** State Key Laboratory of Oral Diseases, National Clinical Research Center for Oral Diseases, Chinese Academy of Medical Sciences Research Unit of Oral Carcinogenesis and Management, West China Hospital of Stomatology, Sichuan University, Chengdu, China

**Keywords:** narrow band imaging, malignant transformation, oral potentially malignant disorders, meta-analysis, systematic review

## Abstract

**Objective:**

Oral potentially malignant disorders (OPMDs) are a spectrum of diseases that harbor the potential of malignant transformation and developing into oral squamous cell carcinoma (OSCC). Narrow band imaging (NBI) has been clinically utilized for the adjuvant diagnosis of OPMD and OSCC. This study aimed to comprehensively evaluate the diagnostic accuracy of NBI for malignant transformations of OPMD by applying the intraepithelial papillary capillary loop (IPCL) classification approach.

**Methods:**

Studies reporting the diagnostic validity of NBI in the detection of OPMD/OSCC were selected. Four databases were searched and 11 articles were included in the meta-analysis. We performed four subgroup analyses by defining IPCL I/II as negative diagnostic results and no/mild dysplasia as negative pathological outcome. Pooled data were analyzed using random-effects models. Meta-regression analysis was performed to explore heterogeneity.

**Results:**

After pooled analysis of the four subgroups, we found that subgroup 1, defining IPCL II and above as a clinically positive result, demonstrated the most optimal overall diagnostic accuracy for the malignant transformation of OPMDs, with a sensitivity and specificity of NBI of 0.87 (95% confidence interval (CI) [0.67, 0.96], *p* < 0.001) and 0.83 [95% CI (0.56, 0.95), *p* < 0.001], respectively; while the other 3 subgroups displayed relatively low sensitivity or specificity.

**Conclusions:**

NBI is a promising and non-invasive adjunctive tool for identifying malignant transformations of OPMDs. The IPCL grading is currently a sound criterion for the clinical application of NBI. After excluding potentially false positive results, these oral lesions classified as IPCL II or above are suggested to undergo biopsy for early and accurate diagnosis as well as management.

## Highlights

1. Narrow band imaging (NBI) is a promising and non-invasive adjunctive tool for identifying malignant transformation of oral potentially malignant disorders and oral cancer.
2. Intra-epithelial papillary capillary loop (IPCL) classification is presently a relatively effective standard for grading the abnormal degree of oral dysplastic lesions upon narrow band imaging detection.
3. After exclusion of false positive findings, oral lesions which is considered as IPCL II or above is recommended to undergo biopsy for avoiding missed diagnosis and timely detection of oral potentially epithelial dysplasia or oral cancer.

## Introduction

Oral potentially malignant disorders (OPMDs), including oral erythroplakia, oral leukoplakia, discoid lupus erythematosus, oral submucous fibrosis, oral lichen planus, actinic cheilitis, and oral chronic candidiasis, are frequently observed in routine clinical practice and have the potential for malignant transformation. For instance, as a typical OPMD, the incidence of oral leukoplakia is 1%–4%, however the malignant risk for localized leukoplakia is 8%–22%, and that for proliferative verrucous leukoplakia is 70%–100% (1). More than 90% of all oral malignancies are oral squamous cell carcinomas (OSCC), which are the most common form of oral cancer (2). Over the past three decades, the 5-year survival rate for OSCC has maintained at around 50% (3). Early detection and treatment of cancer are particularly important for improving the prognosis and survival rate of OSCC (4). Thus, early detection of malignant transformations of OPMDs is of substantial significance, as the treatment is more effective and feasible at the early stage of the disease (5).

Currently, conventional oral examinations (COE) and biopsies of suspicious lesions are routinely applied in dental clinics; however, COE is largely limited to oral medicine specialist clinics that possess higher reliability (6). Pathological examination, as the gold standard for diagnosis, is only adopted in the context of highly suspicious lesions; moreover, it is time-consuming and invasive, which is not suitable for multiple examinations for patients who need long term follow-up. Hence, real-time, non-invasive diagnostic methods are increasingly being adopted by clinicians for less pain, less cost, and more convenience.

**Figure 1:**
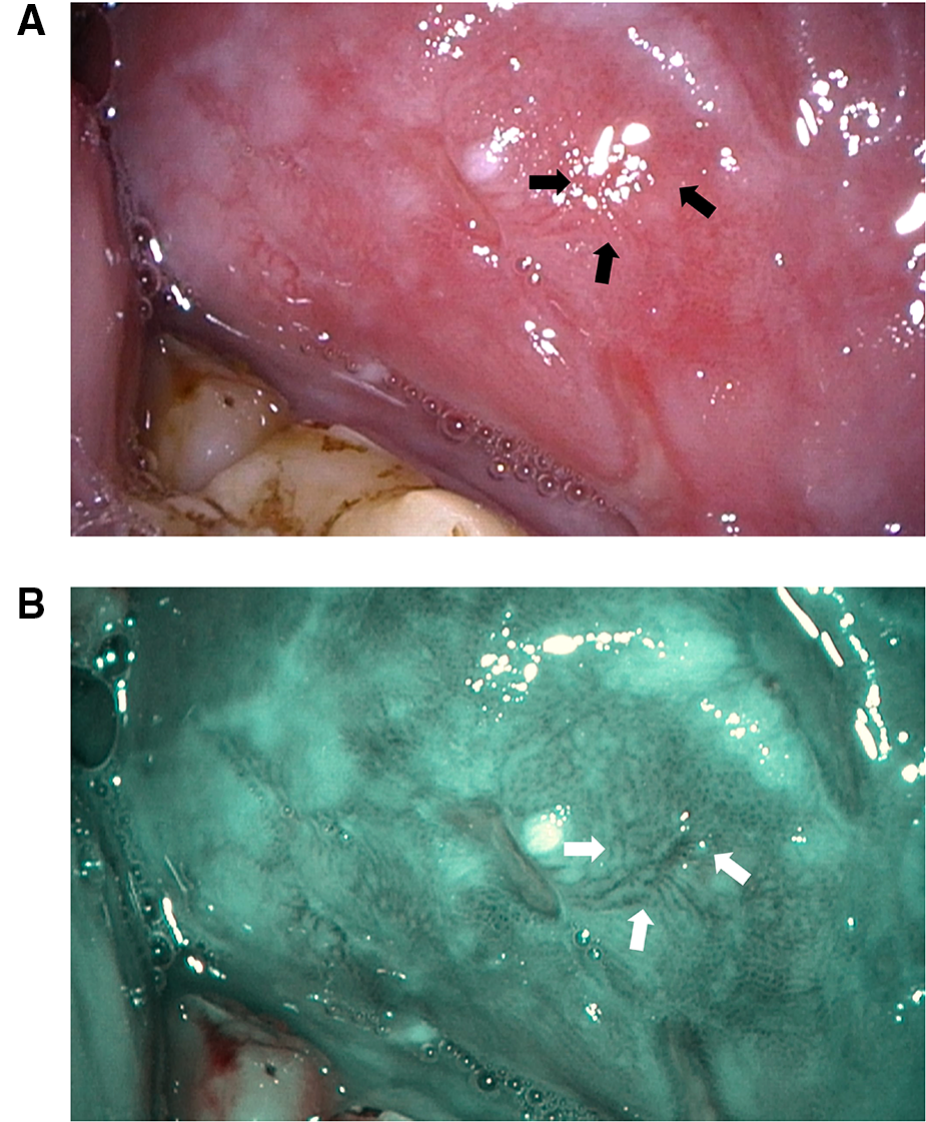
White light (**A**) narrow band (**B**) images of the OPMD lesions which was finally diagnosed as moderate epithelial dysplasia on the left buccal mucosa of one elderly male patient in our clinic. White and black arrows showed the vessels pattern presenting as IPCL III. OPMD, oral potentially malignant disorders; IPCL, intra-epithelial papillary capillary loop.

At present, various non-invasive early diagnostic methods are emerging and gaining significant attention in dental practice (7, 8). Of note, narrow band imaging (NBI; Olympus Medical System Corporation, Tokyo, Japan), as a novel optical method, has recently been applied in the detection of dysplastic or malignant lesions, including those of the oropharynx and nasopharynx, and has exhibited sound performance in diagnosing malignant lesions of the digestive system, such as esophageal cancer (9–11). As for its mechanisms, under NBI mode, blue and green lights whose wavelengths are at 415 and 540 nm, are able to penetrate the mucosal surface and are then absorbed by the surficial blood vessels; thus, the vessels present as dark blue or brown (Figure 1). Hence, compared with white light examination, the contrast of the blood vessels relative to the mucosa is remarkably increased, resulting in a more distinct image of the capillary (12). At present, there are several classification methods for the vessel pattern of oral lesions. The well-recognized classifications of NBI are based on vascular morphology (13), among which the intraepithelial papillary capillary loop (IPCL) approach proposed by Haruhiro Inoueis has been well adopted by scholars for defining oral lesions (14). Specifically, the IPCL classification of abnormal oral squamous epithelium is divided into type I (normal mucosa and regular brown spots), type II (dilation and crossing of blood vessels), type III (elongation and winding of the vessels), and type IV (destruction of the IPCL structure and winding vessels) (15). In addition, multiple studies have indicated that an increasing IPCL pattern might be positively related to incremental epithelial dysplasia (16, 17).

To date, several groups have investigated the effectiveness of NBI in diagnosing OPMD and OSCC, as it can observe alterations of the oral mucosal microvasculature during epithelial carcinogenesis (7, 18). Lin et al. suggested that NBI differs in its ability to identify the IPCL of oral or oropharyngeal mucosa due to the various epithelial structures (19). Moreover, Piazza et al. proposed that NBI has an overall better diagnostic performance for thick, non-keratinized abnormally differentiated epidermis than for thin, non-keratinized epidermis (20). These interesting results motivated us to perform an evidence-based analysis to further assess the value of NBI in diagnosing malignant transformations of OPMD.

Several relevant meta-analyses were first reviewed. One group reported that NBI could improve the diagnostic sensitivity of conventional endoscopic examination in assessing the tissue characteristics of OPMDs; however, no statistical analysis regarding the association between the pathological findings and IPCL patterns was performed (21). In addition, Vu et al. performed a systematic review of NBI for detecting OPMDs, OSCC, and oropharyngeal squamous cell carcinoma, in which a relatively good diagnostic value was indicated; however, the efficacy of NBI was only compared with that of white light examination rather than tissue biopsy (22). In a meta-analysis by Ansari et al., only four eligible studies were included for analysis, in which IPCL was not applied for defining the clinical result but only cancerous tissue was deemed as a positive histological outcome, which implied that NBI has a relatively higher sensitivity in detecting squamous cell carcinoma (23). In 2021, another group conducted a meta-analysis regarding the diagnostic significance of NBI for malignant transformation of OPMDs; however, their study did not consider the association between various grades of epithelial dysplasia and the according IPCL patterns (6).

This systematic review and meta-analysis aim to comprehensively evaluate the diagnostic accuracy of NBI in the malignant transformation of OPMD and explore the potential of employing IPCL classification as the standard for clinical decision-making regarding the execution of surgical biopsy, in which we initially address and analyze the association between IPCL patterns and the pathological results of OPMDs and OSCC. Upon analysis, we concluded that NBI might serve as a promising and non-invasive adjunctive tool for detecting malignant transformations of OPMDs by employing the IPCL grading approach, and that OPMDs identified as IPCL II or above by NBI are highly suggested to undergo biopsy for early detection of epithelial dysplasia and OSCC.

## Methods

### Protocol and registration

The systematic review and meta-analysis were performed based on the Preferred Reporting Items for Systematic Reviews Meta-Analyses (PRISMA) protocols. And the protocol has been registered in the International Prospective Register of Systematic Reviews (PROSPERO) database (CRD42022378948).

### Search strategy

A comprehensive and systematic search of four databases (PubMed, Embase, Web of Science, and Cochrane Library) up to July 2022 was conducted to obtain all relevant studies exploring the precision of NBI in the inchoate detection of OSCC and malignant transformations of OPMDs. The key phrases applied in the search covered “Narrow band imaging,” “Oral potentially malignant disorders,” “Oral premalignant lesions,” “Actinic cheilitis,” “Oral Submucous Fibrosis,” “Lichen planus, Oral,” “Oral leukoplakia,” “Lupus erythematosus, discoid,” “Oral erythroplakia” or “Mouth neoplasm.” The detailed search strategy is presented in Table 1. Other studies, including related articles in the reference lists of the selected literature, were also screened to avoid missing relevant results. In addition, the full texts of all feasible studies were downloaded to verify that they met the inclusion criteria.

**Table 1:** Search strategies of the study.

Databases	Steps	Strategies
PubMed	#1	Squamous Cell Carcinoma of Head and Neck[Mesh] OR oral neoplas*[All fields] OR oral malignan*[All fields] OR oral carcinoma[All fields] OR oral cancer[All fields] OR oral tumor[All fields] OR mouth neoplas*[All fields] OR mouth malignan*[All fields] OR mouth carcinoma[All fields] OR mouth cancer[All fields] OR mouth tumor[All fields]
	#2 #3 #4 #5 #6 #7	Lupus Erythematosus, Discoid[Mesh] OR discoid lupus erythematosus[All fields] OR oral discoid lupus erythematosus[All fields] Leukoplakia, Oral[Mesh] OR oral leukoplakia[All fields] OR oral leucoplakia[All fields] OR oral erythroplakia[All fields] Lichen Planus, Oral[Mesh] OR lichen planus[All fields] OR oral lichen planus[All fields] OR oral lichen[All fields] Oral Submucous Fibrosis[Mesh] OR submucous fibrosis[All fields] AND oral submucous fibrosis[All fields] potentially malignant disorders[All fields] OR potentially malignant oral disorders[All fields] OR oral potentially malignant disorders[All fields] OR potentially malignant oral lesions[All fields] OR precancerous disorders[All fields] OR premalignant oral disorders[All fields] OR precancerous oral disorders[All fields] OR precancerous oral lesions[All fields] OR premalignant oral lesions [All fields] Actinic cheilitis [Supplementary Concept] OR Actinic cheilitis[All fields]
	#8 #9 #10	#1 OR #2 OR #3 OR #4 OR #5 OR #6 OR #7 narrow band imaging[All fields]) OR NBI[All fields] OR narrow-band imaging[All fields] OR narrowband imaging[All fields] #8 AND #9
Embase Web of Science Cochrane Library	#1	“Squamous Cell Carcinoma of Head and Neck” OR “oral neoplasm” OR “oral malignant” OR “oral malignancy” OR “oral carcinoma” OR “oral cancer OR “oral tumor” OR mouth neoplasm” “OR mouth malignant” OR “mouth carcinoma” OR” mouth cancer” OR “mouth tumor”
	#2	“Lupus Erythematosus, Discoid” OR “discoid lupus erythematosus” OR “oral discoid lupus erythematosus”
	#3	“Leukoplakia, Oral” OR oral “leukoplakia” OR “oral leucoplakia” OR “oral erythroplakia”
	#4	“Lichen Planus, Oral” OR “lichen planus” OR “oral lichen planus” OR “oral lichen”
	#5	“Oral Submucous Fibrosis” OR “submucous fibrosis”
	#6	“potentially malignant disorders” OR “potentially malignant oral disorders” OR “oral potentially malignant disorders” OR “potentially malignant oral lesions” OR “precancerous disorders” OR “premalignant oral disorders” OR “precancerous oral disorders” OR “precancerous oral lesions” OR “premalignant oral lesions”
	#7	Actinic cheilitis
	#8	#1 OR #2 OR #3 OR #4 OR #5 OR #6 OR #7
	#9	“Narrow band imaging” OR “NBI” OR “Narrowband imaging” OR “Narrow-band imaging”
	#10	#8 and #9

### Selection criteria

Selected studies conformed to the following inclusion criteria: (1) All suspected OSCC or OPMD samples detected by NBI were from clinical patients, and histopathological results were the gold standard. (2) The classification of NBI patterns was based on IPCL grading criteria. The pathological findings of these samples included mild, moderate, or severe dysplasia and OSCC; other results could be defined as non-dysplastic lesions. (3) Sufficient data were provided to calculate the true positives (TP), true negatives (TN), false positives (FP), and false negatives (FN). (4) English was used as the language.

The exclusion criteria were as follows: (1) animal models; (2) studies in which the diagnostic standard precluded histopathology; (3) case studies; (4) repeated studies; and (5) studies which employed optical evaluation standards other than IPCL.

### Subgroup analysis

Based on the definition of negative results in different studies in terms of various IPCL patterns and pathological grading, the following four subgroups were divided for the meta-analysis. (1) IPCL I was optical-negative; all dysplastic and cancerous lesions were defined as pathologically positive; (2) IPCL I and IPCL II were optical-negative; all dysplastic and cancerous lesions were treated as pathologically positive; (3) IPCL I and IPCL II were optical-negative; only moderate and severe dysplasia and OSCC were regarded as pathologically positive; (4) IPCL I was optical-negative; only moderate and severe dysplasia and OSCC were considered pathologically positive.

### Data extraction and quality assessment

Two reviewers evaluated the risk of bias and applicability concerns of the included studies independently by employing the Quality Assessment of Diagnostic Accuracy Studies II (QUADAS II) tool. Two reviewers independently extracted the information for each article using standardized data extraction tables to evaluate the quality of each article. Disagreements were resolved by discussion. We collected data for the following variables: first author, region, number of patients, sample type, average age, IPCL pattern, and related pathological results. We then extracted or calculated the TP, TN, FP, and FN of each study.

### Statistical analysis

Data processing was carried out using Stata 12.0 (Stata Corporation, College Station, Texas 77, 845 United States). It is common practice to assess the diagnostic utility of particular clinical procedures using the metrics of sensitivity, specificity, positive likelihood ratio (PLR), negative likelihood ratio (NLR), and 95% confidence interval (CI). The aforementioned factors were utilized to evaluate the precision of NBI for diagnosing OPMDs or OSCC. We decided to use a random-effects model (DerSimonian-Laird technique) due to the fact that the research we chose were from various populations. In order to determine the influence of the threshold on the results, summary receiver operating characteristics (SROC) curves were plotted. The lack of a shoulder on the SROC curve suggested that the threshold had no impact on the results. The overall effectiveness of NBI was calculated using the area under the curve (AUC). The DerSimonian-Laird test (Q statistic) and inconsistency index (*I*^2^) statistic were employed to quantify heterogeneity. The degree of heterogeneity was deemed significant when *I*^2^ > 50% and *p* < 0.5, indicating that meta-regression analysis could be utilized to investigate the heterogeneity. To evaluate publication bias, the Deeks funnel chart asymmetry test was applied. Publication bias was considered when the *p*-value was <0.5. We also used Fagan graph analysis, which assesses the correlation between the estimated pre-test probability of the disease, the likelihood ratio of the diagnostic test, and the projected post-test probability of the disease using NBI.

## Results

**Figure 2:**
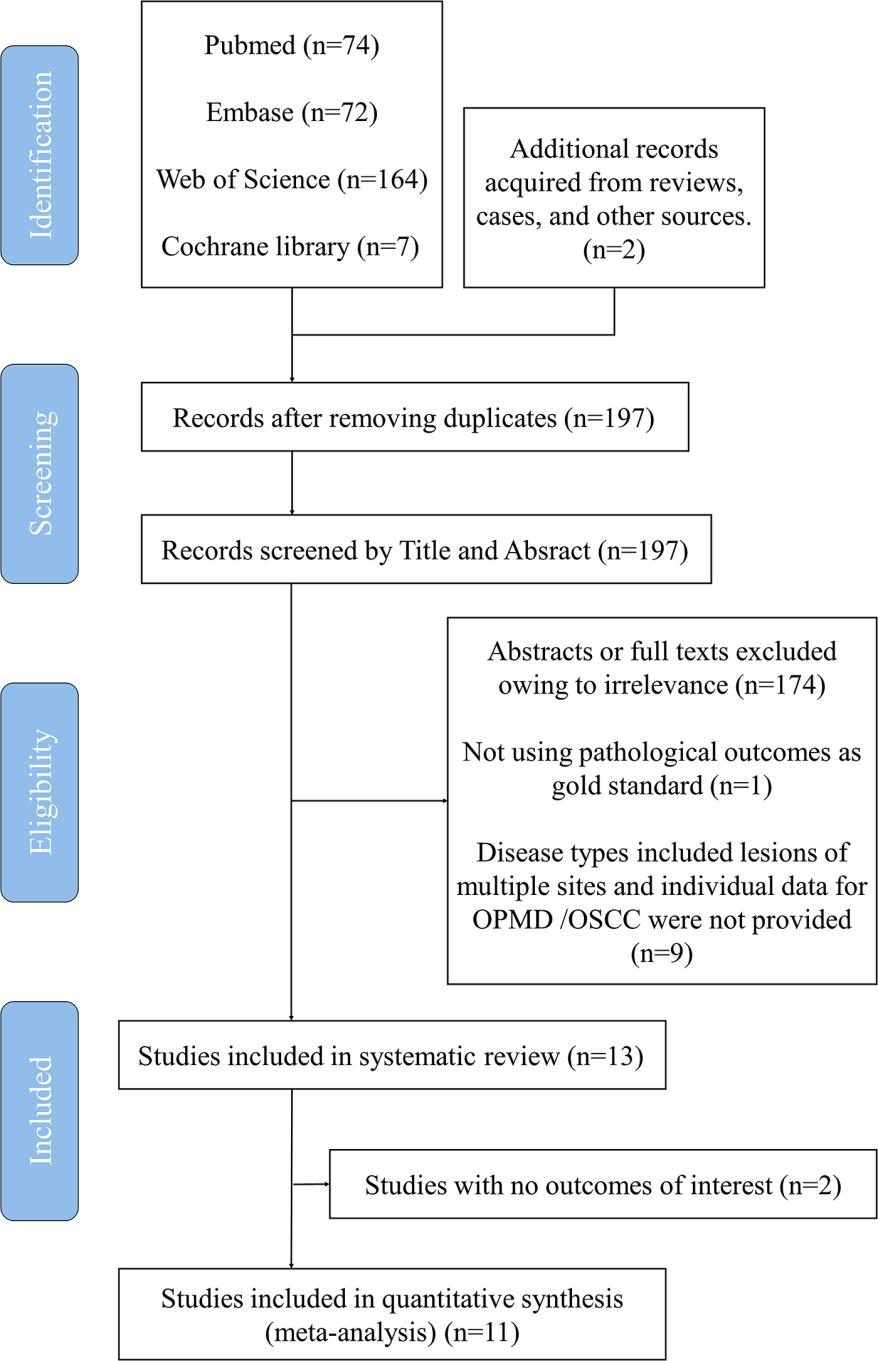
Summary of the search strategy of this study.

A total of 197 related articles were selected after the initial literature search, and 68 articles remained after the removal of duplicates (Figure 2). Thirty-five articles were considered highly relevant after assessing their titles and abstracts. Finally, 13 articles were included in the analysis after reviewing the full texts (Table 2) (15, 20, 24–35). For the meta-analysis, we divided these studies into four subgroups based on different diagnostic criteria for defining positive findings of NBI pattern, and nine, nine, six, and five studies were then pooled respectively to perform the corresponding analysis.

**Table 2:** Summary of the eligible studies applying NBI for detecting the malignant transformation of OPMD and OSCC.

Author, year	Region	Mean Age	Sample type	Diagnostic criterion	Biopsy negative criterion	TP	FP	FN	TN	SEN (95%CI)	SPE (95%CI)	Quadas-II
Shih-Wei Yang, 2011 (15)	China	50.96 ± 10.25	Oral Leukoplakia	histopathology	N	101	4	10	50	0.910 (0.841,0.956)	0.926 (0.821,0.979)	7
Shih-Wei Yang, 2011 (27)	China	52.60 ± 10.86	Oral Leukoplakia	histopathology	N	25	6	26	103	0.490 (0.348,0.634)	0.945 (0.884,0.980)	7
Shih-Wei Yang, 2012 (28)	China	52.15 ± 10.75	Oral Leukoplakia	histopathology	N	180	31	60	137	0.750 (0.690,0.803)	0.815 (0.748,0.871)	7
T. Shibahara, 2013 (31)	Japan	U	OSCC/OPMD	histopathology	N&L	48	8	4	77	0.923 (0.815,0.979)	0.906 (0.823,0.958)	6
Shih-Wei Yang, 2014 (29)	China	57.9 ± 16.7	Oral Chronic Non-healing Ulcers	histopathology	N&L	15	4	1	43	0.938 (0.698,0.998)	0.916 (0.796,0.976)	7
Shih-Wei Yang, 2015 (30)	China	54.6 ± 11.2	Oral Erythroplakia	histopathology	N	60	6	0	6	1.000 (0.940,1.000)	0.500 (0.211,0.789)	7
AN Vu, 2015 (21)	Australia	58.58 ± 9.42 62.09 ± 11.12	OPMD	histopathology	N	60	20	8	5	0.882 (0.781,0.948)	0.200 (0.068,0.407)	7
Giulia Ottaviani, 2016 (26)	Italy	U	OSCC	histopathology	N	23	0	0	68	1.000 (0.852,1.000)	1.000 (0.947,1.000)	6
Pizza C, 2016 (19)	Italy	65	OSCC/OPMD	histopathology	N	80	6	10	32	0.889 (0.805,0.945)	0.842 (0.804,0.871)	U
Aparaajita Upadhyay, 2019 (25)	India	U	Oral Malignant Lesions	histopathology	N	31	0	6	1	0.838 (0.680,0.938)	1.000 (0.025,1.000)	5
Agostino Guida, 2019 (22)	Italy	61 ± 13.7	OSCC/OPMD	histopathology	N	59	19	7	11	0.894 (0.794,0.956)	0.367 (0.199,0.561)	6
Alberto Deganello, 2020 (23)	Italy	68	Oral Lichen Planus	histopathology	N&L	5	2	0	49	1.000 (0.478,1.000)	0.961 (0.865,0.995)	6
Airi Ota, 2022 (32)	Jagan	U	OSCC/OPMD	histopathology	N	U	U	U	U	1.000 (0.661,1.000)	0.809 (0.667,0.909)	U

NBI, narrow band imaging; OSCC, oral squamous cell carcinoma; OPMD, oral potentially malignant disorders; U, unknown; N, non-dysplasia; L, low-grade dysplasia; SEN, sensitivity; SPE, specificity; CI, confidence interval; TP, true positive; FP, false positive; TN, true negative; FN, false negative.

### Study characteristics

The 13 studies were published between 2010 and 2022, with 1,648 participants from Australia, China, India, Italy, and Japan. All studies included were case-control clinical trials, and quality assessment was evaluated using the QUADAS II tool (Table 3). All studies reported the sensitivity and specificity of NBI for detecting suspicious premalignant and malignant oral lesions. Although Pizza et al. mentioned the significance and value of IPCL in detecting OPMDs along with OSCC in their article, the statistical analysis of their results was not based on the grading of IPCL patterns; thus, it was excluded from the analysis. In addition, owing to the different study types, the study by Ari Ota et al. was not included in the meta-analysis. Descriptive analysis of these two excluded studies was then conducted.

**Table 3:** Assessment of studies regarding diagnostic performance of NBI by using the QUADAS-2 tool.

Study	Risk of bias				Applicability concerns		
	Patient selection	Index test	Reference standard	Flow and timing	Patient selection	Index test	Reference standard
Shih-Wei Yang, 2012 (15)	Unclear	Low	Low	High	Low	Low	Low
Shih-Wei Yang, 2012 (27)	Unclear	Low	Low	High	Low	Low	Low
Shih-Wei Yang, 2012 (28)	Unclear	Low	Low	High	Low	Low	Low
T. Shibahara, 2013 (31)	Unclear	Unclear	Low	Low	Low	Unclear	Low
Shih-Wei Yang, 2014 (29)	Unclear	Low	Low	High	Low	Unclear	Low
Shih-Wei Yang, 2015 (30)	Unclear	Low	Low	High	Low	Low	Low
AN Vu, 2015 (21)	High	Low	Low	High	Low	Low	Low
Giulia Ottaviani, 2016 (26)	High	Low	Low	Low	High	Low	Low
Aparaajita Upadhyay, 2019 (19)	High	Low	Low	Unclear	High	Low	Low
Agostino Guida, 2019 (22)	Unclear	High	Low	Low	Low	Low	Low
Alberto Deganello, 2020 (23)	Low	High	Low	High	Low	Unclear	Low

Among these 11 eligible studies, lesions with no dysplasia were deemed as negative pathological results in 8 studies and mild epithelial dysplasia or lower was defined as a negative finding in 3 articles. Regarding IPCL findings, 10 articles treated lesions of IPCL I as a negative result, and 1 study defined the negative finding as a lesion belonging to IPCL II or I. Accordingly, these 11 studies were divided into four subgroups to conduct further analysis (Table 4).

**Table 4:** Summarized diagnostic value of NBI for OPMD and OSCC within various subgroups.

Subgroup analysis	Studies	Participants	Sensitivity	Specificity	Diagnostic Odds Ratio	Positive likelihood ratio	Negative likelihood ratio	Area Under Curve
Subgroup 1	9	1179	0.87 (0.67, 0.96)	0.83 (0.56, 0.95)	87.40 (8.62, 886.50)	7.32 (1.60, 33.47)	0.08 (0.02, 0.29)	0.92 (0.89, 0.94)
Subgroup 2	9	1232	0.58 (0.19, 0.89)	0.95 (0.85, 0.99)	28.38 (7.18, 112.11)	12.42 (4.72, 32.68)	0.44 (0.16, 1.18)	0.94 (0.91, 0.96)
Subgroup 3	6	946	0.69 (0.47, 0.85)	0.97 (0.88, 0.99)	63.54 (20.17, 200.19)	20.14 (6.25, 64.85)	0.32 (0.17, 0.60)	0.93 (0.90, 0.95)
Subgroup 4	5	890	0.97 (0.82, 0.99)	0.55 (0.23, 0.83)	36.21 (5.38, 243.74)	2.14 (1.02, 4.49)	0.06 (0.01, 0.33)	0.92 (0.90, 0.94)

NBI, narrow band imaging; OSCC, oral squamous cell carcinoma; OPMD, oral potentially malignant disorders.

### Diagnostic accuracy of NBI for OPMD and OSCC

**Figure 3:**
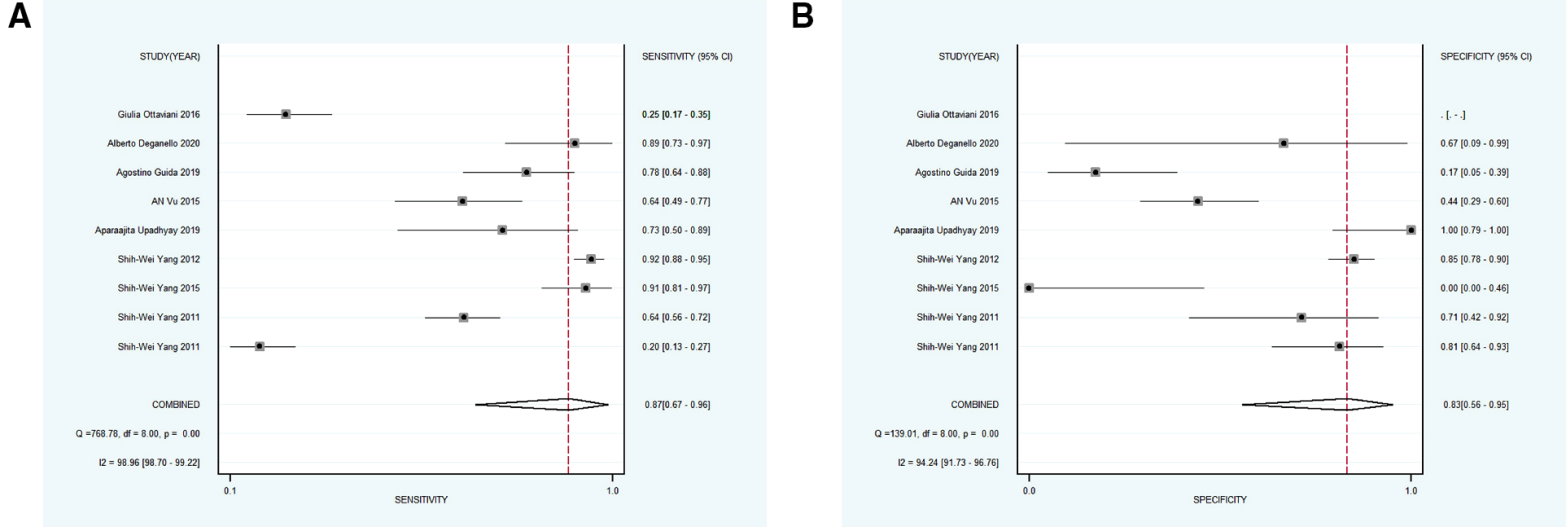
(**A,B**) forest plot of sensitivity and specificity of subgroup 1 regarding the diagnostic accuracy of NBI for the malignant transformation of OPMD.

**Figure 4:**
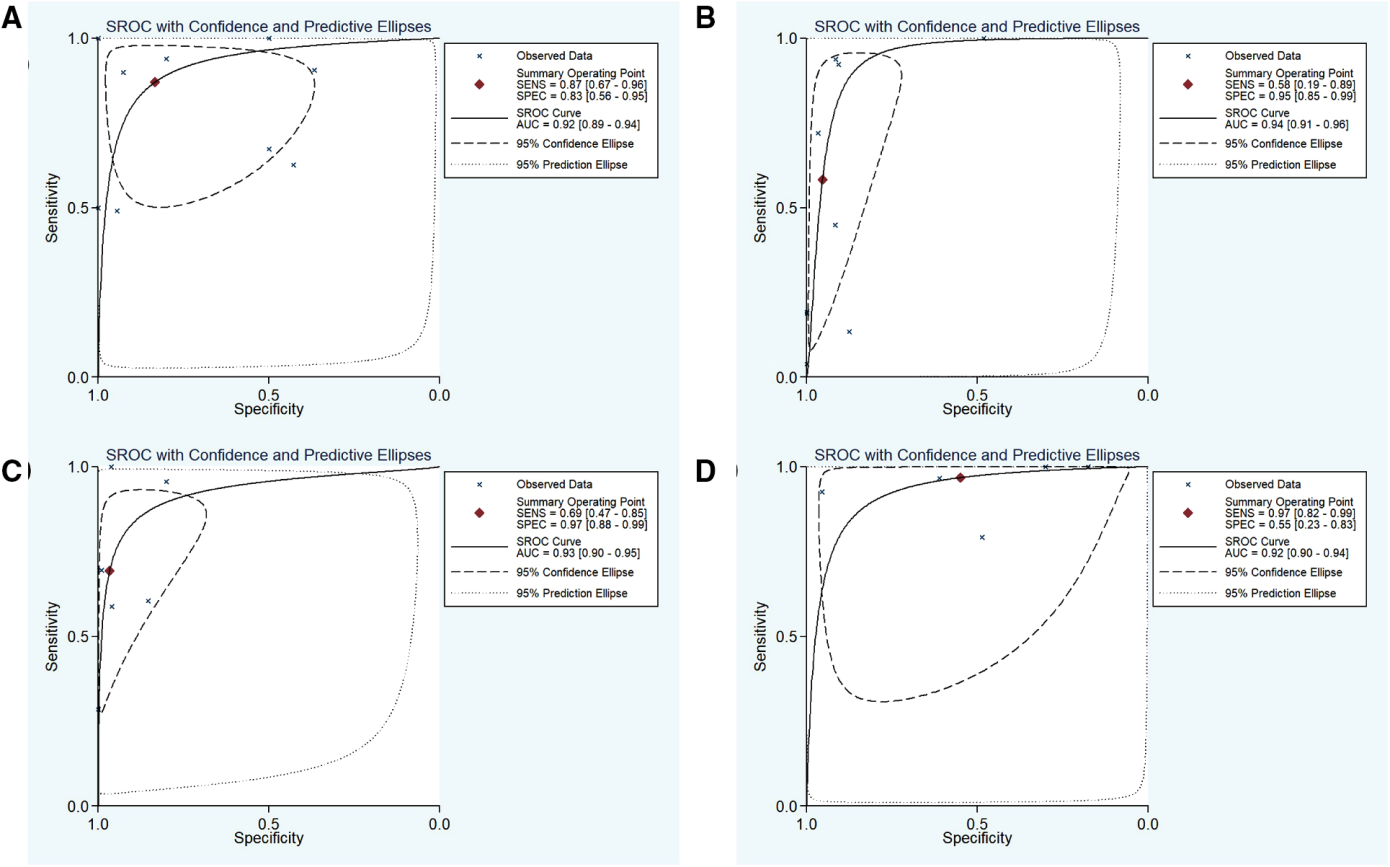
(**A–D**) SROC plots of subgroup 1–4, respectively. SROC, summary receiver operating characteristics.

Nine studies with 1,179 participants were categorized into subgroup 1 based on their characteristics, the coalescent sensitivity and specificity results for NBI in the diagnosis of OPMD and OSCC were 0.87 [95% CI (0.67, 0.96), *p* < 0.001, *I*^2^ = 98.96] and 0.83 [95% CI (0.56, 0.95), *p* < 0.001, *I*^2^ = 94.24], and the pooled diagnostic odds ratio (DOR) was 87.40 (95% CI, 8.62, 886.50). In addition, the PLR and NLR were 7.32 (95% CI, 1.60, 33.47) and 0.08 (95% CI, 0.02, 0.29), respectively. The AUC showed an optimal diagnostic value of 0.92 (95% CI, 0.89, 0.94) (Figures 3A,B, 4A).

**Figure 5:**
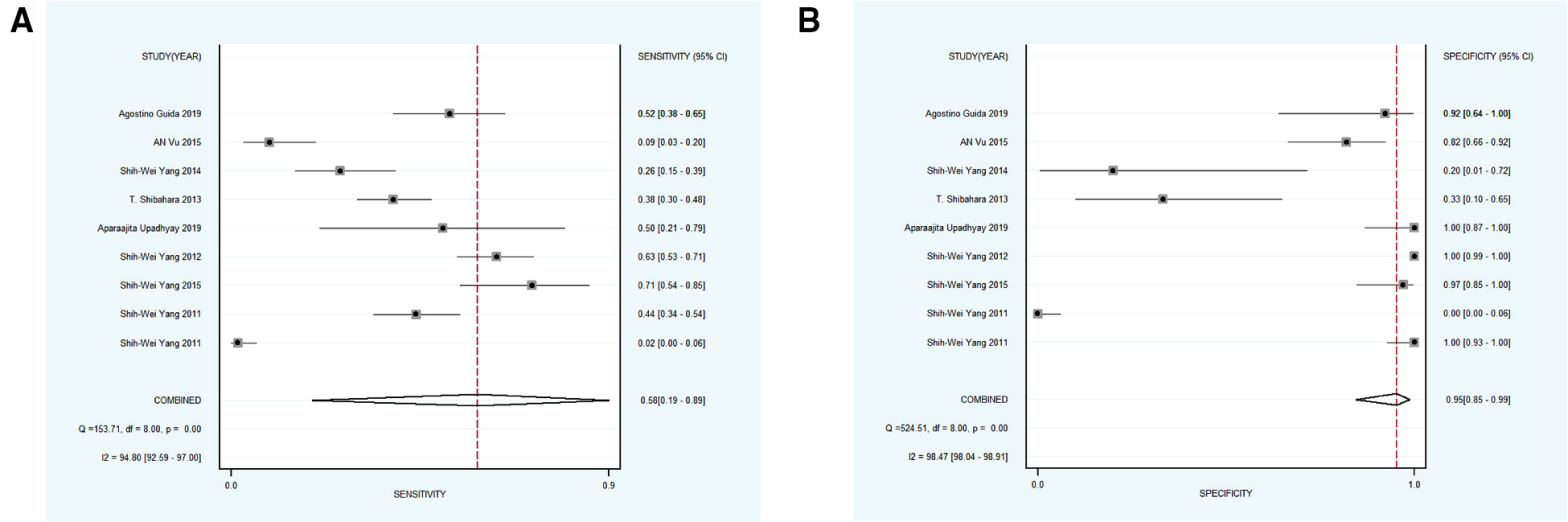
(**A,B**) forest plot of sensitivity and specificity of subgroup 2 regarding the accuracy of NBI for identifying the malignant transformation of OPMD.

Subsequently, 9 studies with 1,232 participants were included into subgroup 2, the merged sensitivity and specificity results for NBI were 0.58 [95% CI (0.19, 0.89), *p* < 0.001, *I*^2^ = 94.80] and 0.95 [95% CI (0.85, 0.99), *p* < 0.001, *I*^2^ = 98.47], and the pooled DOR was 28.38 (95% CI, 7.18, 112.11), the PLR and NLR were calculated as 12.42 (95% CI, 4.72, 32.68) and 0.44 (95% CI, 0.16, 1.18), respectively. In addition, the AUC was 0.94 (95% CI, 0.91, 0.96), representing a sound diagnostic value (Figures 4B, 5A,B).

**Figure 6:**
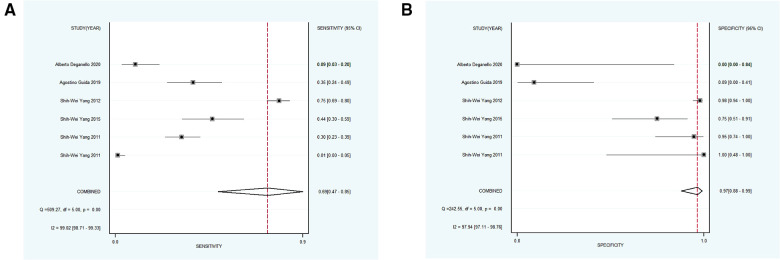
(**A,B**) forest plot of sensitivity and specificity of subgroup 3 pertaining the diagnostic accuracy of NBI for the malignant transformation of OPMD.

Next, 6 studies with 946 participants which met the standard were classified as subgroup 3, the merged sensitivity and specificity results for NBI were 0.69 [95% CI (0.47, 0.85), *p* < 0.001, *I*^2^ = 99.02] and 0.97 [95% CI (0.88, 0.99), *p* < 0.001, *I*^2^ = 97.94], the pooled DOR was 63.54 (95% CI, 20.17, 200.19), and the PLR and NLR were 20.14 (95% CI, 6.25, 64.85) and 0.32 (95% CI, 0.17, 0.60), respectively. The AUC was 0.93 (95% CI, 0.90, 0.95), representing a relatively high diagnostic value (Figures 4C, 6A,B).

**Figure 7:**
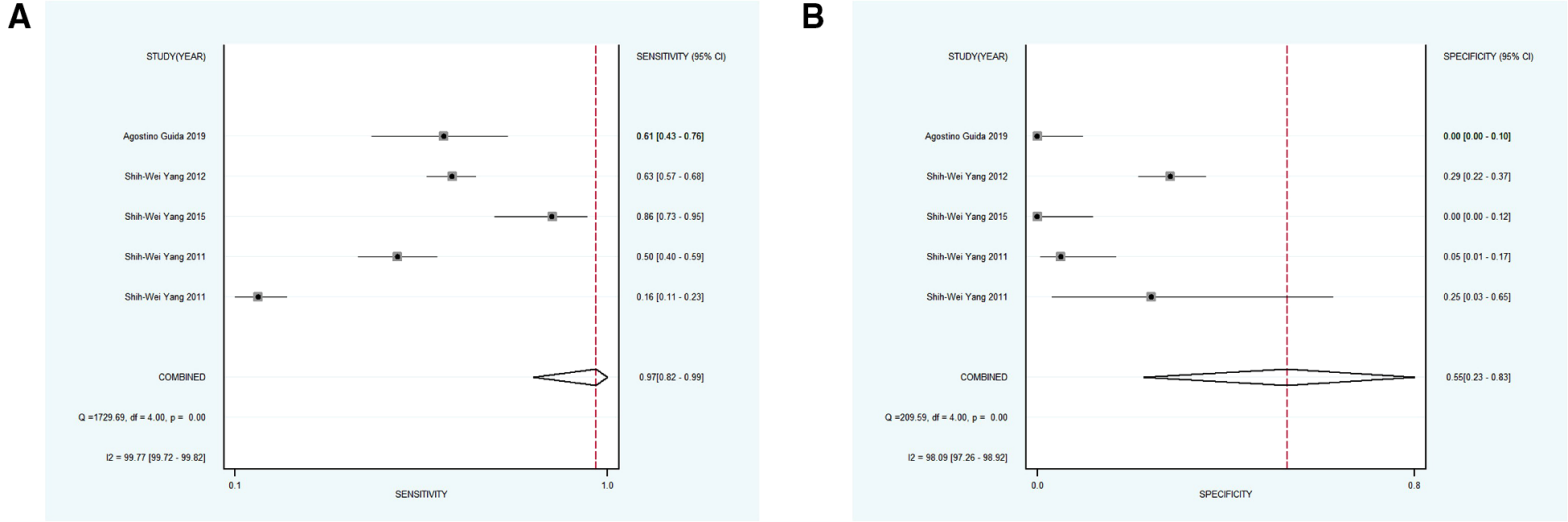
(**A,B**) forest plot of sensitivity and specificity of subgroup 4 regarding the detecting value of NBI for the malignant transformation of OPMD. OPMD, oral potentially malignant disorder.

Using varying standards of classification, 5 studies with 890 participants were then divided into subgroup 4, the pooled sensitivity and specificity results for NBI were 0.97 [95% CI (0.82, 0.99), *p* < 0.001, *I*^2^ = 99.77] and 0.55 [95% CI (0.23, 0.83), *p* < 0.001, *I*^2^ = 98.09], the coalescent DOR was 36.21 (95% CI, 5.38, 243.74), and the PLR and NLR were 2.14 (95% CI, 1.02, 4.49) and 0.06 (95% CI, 0.01, 0.33), respectively. Moreover, the AUC represented a relatively sound diagnostic value of 0.92 (0.90, 0.94) (Figures 4D, 7A,B).

### Publication bias

**Figure 8:**
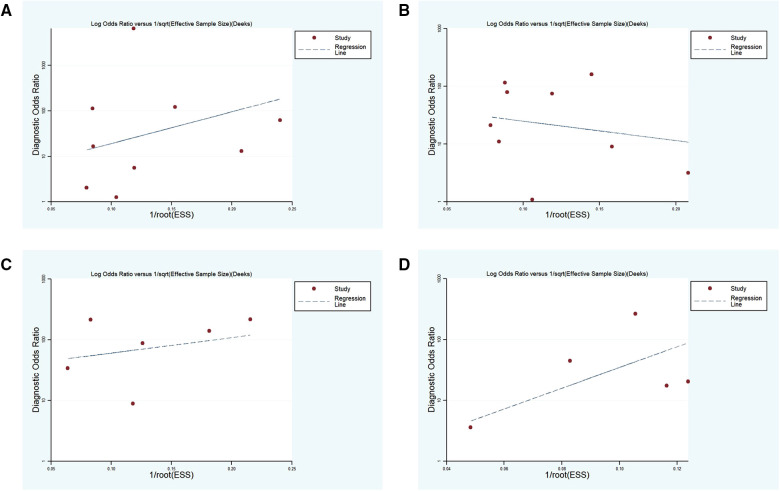
(**A–D**) funnel plots of subgroup 1–4, respectively.

Deeks' funnel chart asymmetry test was adopted to analyze the above four subgroups, and no significant publication bias was revealed (Figure 8).

### Heterogeneity test

The forest plot of sensitivity and specificity for relevant studies in the four subgroups revealed the heterogeneity of the aforementioned studies. After dividing the articles based on various regions, grades of QUADAS II disease type, and number of participants, meta-regression was utilized to deal with the heterogeneity. The number of participants and results of the QUADAS II grading system might be the source of heterogeneity (Supplementary Table S1).

### Fagan analysis

The Fagan analysis showed that the pre-test probability was 30%. In subgroup 1, the positive and negative post-test probabilities were 69% and 6%, respectively. For subgroup 2, under the same circumstances, the probability of a positive post-test was 84% and the negative post-test probability was 16%. The parameters for subgroup 3 were 90% and 12%, respectively. Finally, the probability of a positive post-test was 48% and the negative post-test probability was 2% for subgroup 4. Each subgroup presented a significant difference; however, the post-test probability was affected by the incidence rate (Supplementary Figure S1). Considering the significant variations in the incidence of OPMD in different regions and ethnicities, the post-test probability could not be used as an intuitive criterion for diagnostic efficacy.

## Discussion

This is a comprehensive systematic review and meta-analysis investigating the diagnostic accuracy of NBI in the early screening of malignant transformations of OPMDs by associating the pathological results with the IPCL classification proposed by Inoueis et al. As various standards were defined for negative findings, for the IPCL patterns, and for histological grading in the 11 eligible studies, we divided the studies into four subgroups. Overall, NBI has demonstrated good diagnostic value in detecting oral lesions with dysplasia or cancer, especially when we adopted the diagnostic standard for subgroup 1, which achieved the most optimal diagnostic efficacy. Therefore, defining the IPCL I as negative seems to serve as the most promising criterion to reveal lesions with any degree of dysplasia or cancer. To avoid unnecessary delay in treatment, dental clinicians should consider the application of biopsy after detecting abnormal patterns of NBI, including IPCL II and higher levels. Moreover, the Fagan chart analysis based on subgroup 1 showed that at low clinical suspicion (post-test probability of response of 30%), the post-test probability of a negative malignant result was 6%, which can be considered sufficient to rule out the possibility of epithelial dysplasia, and also indicates that lesions detected as IPCL I may not require biopsy. In agreement with this, its low NLR might also allow the needlessness of biopsy in cases with IPCL I. Additionally, our results showed lower sensitivity in subgroups 2 and 3, further suggesting that the level of IPCL II should not be ignored when considering biopsy. Moreover, the lower specificity of subgroup 4 also suggests that IPCL II or higher might identify lesions with mild epithelial dysplasia. Thus, in summary, the strengths of this study are as the following: first, we comprehensively performed a systematic evaluation and meta-analysis of the literatures regarding accuracy of NBI in diagnosing OSCC and malignant transformation of OPMDs; second, we have performed subgroup analysis to achieve more precise results regarding the efficacy of NBI since various criteria are defined as positive clinical finding of NBI in these include studies; third, based on this study, we suggested that OPMD lesions harboring alterations of IPCL II or above for which false positive result has been excluded should be recommended to undergo biopsy due to the potential presence of epithelial dysplasia or cancer, and this finding would serve as an important clue in clinical practice.

NBI is a safe, well-tolerated, and fast method to serve as a recommended part of the follow-up protocol for patients with OPMD or OSCC. Yang et al. reported that NBI increased the specificity of diagnosis by more than 30% compared to broadband white light; when both are applied in conjunction, the sensitivity can be enhanced significantly(35). Moreover, Takano reported that IPCL II was an important signal of vascular changes in cancer, which is consistent with our findings; however, Kim et al. found that classifications III and IV are more likely to indicate a higher diagnostic accuracy of dysplasia and cancer (21, 29). The above divergence may be related to the small sample size and distinct standards for IPCL classification; notably, all these studies consistently implied the diagnostic efficacy of NBI for OPMD and OSCC. However, large-sample clinical trials are required to further validate the capacity of NBI to detect OPMD or OSCC and better guide clinical practice.

As mentioned above, inchoate screening for OPMD or OSCC in general dental clinics is crucial to improve disease prognosis. However, in daily practice, NBI is rarely applied in general clinics, which correlates with a lack of training and promotion. In the meta-analysis by Ansari et al., NBI was more likely to be applied in specialist clinics because the precise judgment of the result is largely dependent on the operators' skills (23). Although conflicting results have been reported regarding the efficacy of executing appropriate training to improve the diagnostic accuracy of NBI in dental clinics, it is of great significance for general dentists to receive proper and strict training regarding the use of NBI and interpretation of the IPCL patterns, thus enhancing the detection rate of suspicious oral lesions (36). In addition, with the popularization of computer technology, increasing attention is being paid to the combination of diagnostic technology with algorithms or artificial intelligence, which might be of high value in augmenting the diagnostic potential of NBI for identifying malignant transformations of OPMDs in the near future (37–39).

Although NBI has proven to be an efficient diagnostic tool, scholars have also observed some of its drawbacks. NBI is sometimes regarded as less accurate in the presence of certain epithelial types; for instance, thick and keratinized mucosal areas block the underlying blood vessels from absorbing light. To offset the shortage of the above-mentioned condition, some studies have applied the vascular pattern around the lesion as an evaluation indicator, but it is doubtful that these peripheral features may not reflect the real conditions hidden under the hyperkeratotic lesion; for instance, approximately 28% of thick homogeneous leukoplakia surrounded by type I IPCL are actually accompanied by dysplasia (20). Second, bleeding in the visual field during NBI may interfere with the mucosal blood vessel pattern (40). Third, diffusing and severe chronic inflammation of oral lichen planus generally leads to abnormal vascular patterns (33). Finally, visualization of the vascular pattern may also be confusing in oral ulcers owing to the presence of fibrin slough or pseudomembrane. These reasons are responsible for FP and FN. In light of these false results, clinicians should not only strictly obtain the diagnosis under the IPCL classification, but also conduct regular follow-up of NBI examinations based on clinical experience to reduce the possibility of misdiagnosis. Cozzani et al. indicated that lesions should be re-evaluated one month later with NBI after screening to avoid false positive results (41). Therefore, it is highly suggested that OPMD lesions detected as IPCL II or above should be evaluated for the possibility of FP before performing biopsy.

The present study had a certain degree of heterogeneity. Studies from Chinese sources were from the same research group, which leads to potential bias owing to the group's subjective judgment in diagnosis. Moreover, only oral leukoplakia could be separated for further analysis, and the lack of classification of other premalignant lesions might also have contributed to the heterogeneity. Moreover, eight studies were clinical trials with small samples, which, to a certain extent, induced heterogeneity.

The present study has several limitations. First, the included studies were limited to English language publications. Second, none of the included studies clearly illuminated the study design. Third, in some studies, the blinding of pathologists from the results of the NBI assessment was unclear. Finally, no study included long-term follow-up of patients who were considered positive after NBI examinations. We recommend that clinical researchers should design more rigorous randomized controlled trials, including a large sample size, perform NBI examinations for long-term follow-up, and evaluate the contribution of NBI in the context of reducing the incidence of OSCC.

## Conclusion

NBI is an effective non-invasive diagnostic tool with relatively high accuracy. IPCL classification is presently an optimal approach in the judgment of NBI patterns of OPMD and OSCC, providing evidence for early detection of malignant transformations of OPMDs. Moreover, we recommend “IPCL I as optical negative, non-dysplasia, and non-cancer as pathological negative” for guiding the next step of tissue biopsy; to be specific, those lesions, excluding false positive results, which were classified as IPCL II or above, are highly recommended to undergo biopsy for safety monitoring. In the future, more large-sample studies are warranted to confirm the accuracy of NBI in the detection of OPMD and OSCC, NBI should be promoted in general dental clinics upon sufficient training of primary care doctors, and the combination of NBI with advanced technology such as computer-aided diagnostic systems should be evaluated.

## Data Availability

The original contributions presented in the study are included in the article/**Supplementary Material**, further inquiries can be directed to the corresponding author/s.
