# Supplementary material for: Accuracy of narrow band imaging for detecting the malignant transformation of oral potentially malignant disorders: A systematic review and meta-analysis
Source: Front Surg. 2023 Jan 6;9:1068256. doi: 10.3389/fsurg.2022.1068256 (PMC9857777; doi:10.3389/fsurg.2022.1068256)
Supplement: Supplementary file 1 [file Table1.docx]

**Supplemental Table 1**

**Meta-Regression of subgroups which include studies≥8**

|  | Subgroup 1 | Subgroup 2 |
| --- | --- | --- |
|  | *p* | *p* |
| Disease type^*^ | 0.1747 | 0.7527 |
| Regions^#^ | 0.1855 | 0.8268 |
| Sample size^+^ | 0.0089 | 0.4706 |
| Quadas-II^※^ | 0.0228 | 0.8245 |

*: The disease type was distinguished by whether the subjects were diagnosed as OSCC.

#:The regional classification was Asian/ non-Asian.

+:The sample size of </≥80 was defined as the distinction line.

※Statistics of the QUADAS II score was performed on the studies in which the total score of QUADAS II was ≥7.
