# Supplementary figures and images for: Accuracy of narrow band imaging for detecting the malignant transformation of oral potentially malignant disorders: A systematic review and meta-analysis
Source: Front Surg. 2023 Jan 6;9:1068256. doi: 10.3389/fsurg.2022.1068256 (PMC9857777; doi:10.3389/fsurg.2022.1068256)

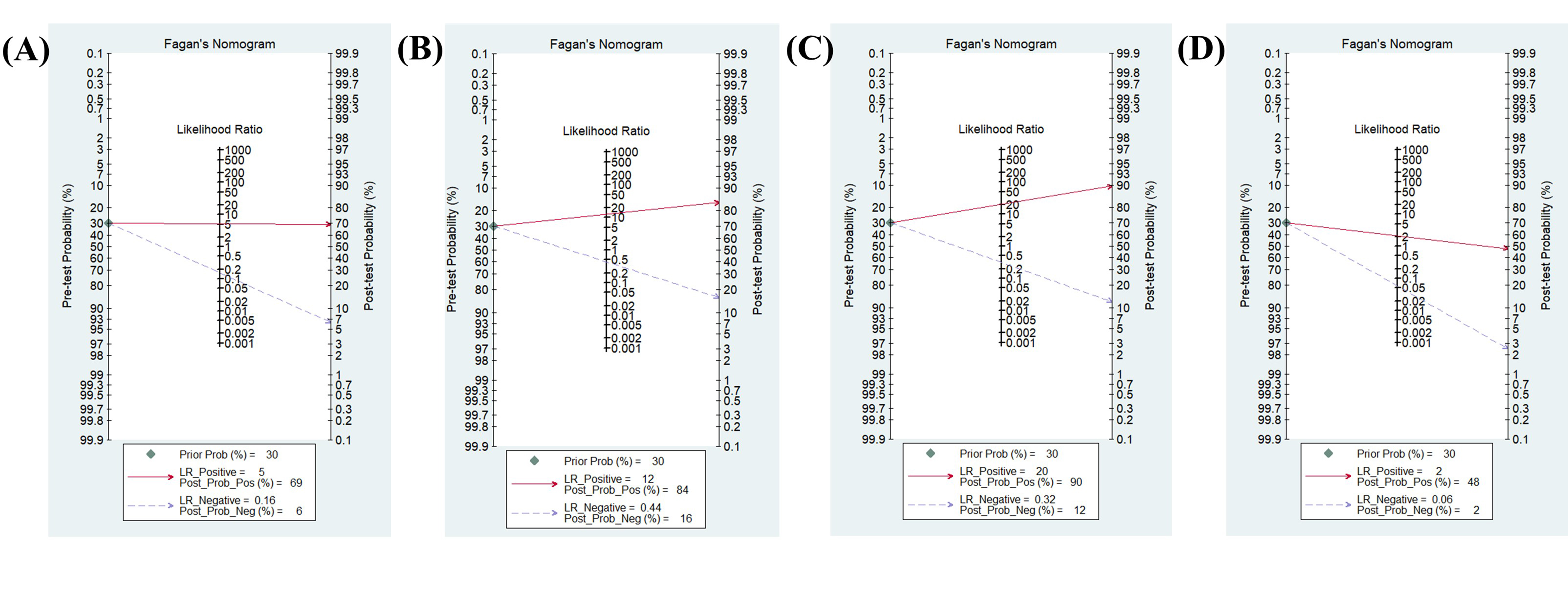

Supplement: Supplementary file 2 [file Image1.jpeg]
